# Selective Aqueous Chemical Solution Deposition Using Patterned Self-Assembling Monolayers

**DOI:** 10.1021/acsomega.5c11372

**Published:** 2026-03-30

**Authors:** Karola Neeleman, Hung Quoc Nguyen, Daniel Rettenwander, Julia Glaum, Mari-Ann Einarsrud

**Affiliations:** Department of Materials Science, 8018 NTNU Norwegian University of Science and Technology, Trondheim 7034, Norway

## Abstract

Bottom-up fabrication techniques, such as Selective Area Deposition, have gained attention in the field of nanotechnology due to their ability to unlock novel structures as well as improve sustainability in fabrication; however, these techniques are incompatible with sustainable chemical solution deposition techniques. In this paper, a novel selective area deposition technique is introduced that is compatible with chemical solution deposition. This new technique combines the utility of selective area deposition with established solution-based film deposition. Using the deposition of barium titanate as a model system, an aqueous precursor solution with high selectivity for the hydrophilic regions was successfully deposited on a hydrophobic/hydrophilic patterned Pt/Si substrate. The substrate was coated with a hydrophobic 1-octadecanethiol self-assembling monolayer that was selectively oxidized by exposure to UV-generated reactive oxygen species. The oxidized molecules were dissolved in ethanol to recover the hydrophilic Pt surface before depositing the precursor solution. The spin-coated precursor solution showed great selectivity for the exposed regions of the substrate after a 25 min UV exposure. The deposited barium titanate test structures were recognizably stripe-like down to a feature size of 27.8 μm, and the smallest recognizable feature size achieved was 15.6 μm, offering potential as a sustainable micropatterning process for films deposited by CSD.

## Graphical abstract

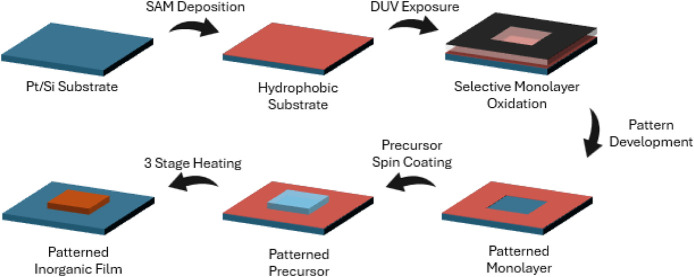

## 1 Introduction

Bottom-up fabrication techniques of nanostructured materials are of utmost importance in the continued development of micro- and nanotechnologies. Bottom-up fabrication techniques use atoms, ions or molecules as building blocks that are assembled to form the desired structures by controlling the chemical interactions between them. In top-down manufacturing, in contrast, the desired structures are shaped from a bulk material by selectively removing undesired material, usually by etching. Bottom-up fabrication techniques are of interest for several reasons, including access to complex 3D structures,^1^ reduced material and energy cost,^2^ and compatibility with etch sensitive materials.^3^ Thin film technology heavily depends on the top-down litho-etch fabrication process to fabricate functional devices from films. Bottom-up thin film fabrication, also referred to selective area deposition, selective area epitaxy, or selective area growth,^2^ can, among others, reduce contamination and provide compatibility with etch sensitive substrates.

Research into selective area deposition has focused primarily on vapor-phase deposition techniques, such as atomic layer deposition^1,4^ and chemical vapor deposition^5,6^ by manipulating the adsorption behavior of vapor-phase species on the substrate. Recent advancements in vapor phase selective area deposition have allowed for the deposition of structures with a width of <100 nm,^7,8^ multilayer compositions,^8^ and complex 2D^7^ and 2.5D^9^ shapes. However, these techniques are incompatible with solution-based thin film deposition techniques such as chemical solution deposition (CSD). CSD is used to deposit a wide range of oxide materials,^10–12^ offering excellent control over stoichiometry for doped materials and complex oxides, high scalability, and simple, low-cost fabrication. In recent years, research into solution chemistry has unlocked synthesis routes using solvents such as ethylene glycol^13^ and water^14^ instead of more hazardous organic solvents, greatly improving the sustainability of CSD processes. At present, no selective area deposition process that is compatible with aqueous CSD of thin films has been published. Such a deposition process would combine the advantages of aqueous CSD and selective area deposition, and deposit high quality patterned oxide films in a low-cost, highly scalable, and sustainable manner. To achieve this, a new functionalization process is required to induce selectivity during the deposition process. Self-assembling monolayers (SAMs) are a powerful tool for functionalizing surfaces, as they can be used to apply a wide variety of properties to substrates and can be patterned by, e.g., inkjet printing,^13^ soft-lithography,^1^ and photolithography.^15–18^ Additionally, the chemical bond between SAMs and the substrate makes these monolayers more robust than physisorbed films such as Langmuir–Blodgett films,^19^ making them more stable under process conditions. Patterned SAMs have previously been demonstrated to be capable of manipulating the flow behavior of liquids on a surface, both under static;^20,21^ and dynamic^22^ conditions, making them a promising tool for selective CSD. Similar processes combining patterned SAMs and solution-based deposition achieved structures as small as 4 μm.^23^ However, these processes either use hydrophilic SAMs to improve the selectivity during deposition,^24,25^ breaking up the substrate–film interface, or require an additional delamination step to remove unwanted material,^23,26^ resulting in a process closer to liftoff patterning than selective area deposition. Higher selectivity has been achieved in aqueous chemical bath deposition, but this process requires long deposition times and results in low density films.^27,28^ By using aqueous CSD, we seek to selectively deposit high density oxide films without the need for any shaping processes post deposition.

Here we report proof-of-concept for a selective area deposition method compatible with aqueous CSD using SAMs. Deposition of BaTiO_3_ (BTO) films on patterned platinized silicon substrates showed great selectivity for the patterned regions of the substrate, resulting in microscale patterning of the deposited film. Control over the surface properties of the substrate during each step of the process proved critical for achieving sufficient selectivity during deposition. Confining the solution on the hydrophilic regions of a patterned substrate significantly alters the behavior of the CSD precursor solution during the spin coating process. The features and limitations introduced by this novel process were investigated, and their influence on the morphology of the deposited structures will be discussed.

## 2 Methods

### 2.1 Sample Preparation

Pt/Si substrates (625 μm Si wafer with 100 nm Pt, purchased from SINTEF, Oslo, Norway) were functionalized by solution phase deposition of 1-octadecanethiol. Before deposition, the substrates were cleaned with oxygen plasma (Femto, Diener Electronic) for 2 min using a 50/50 mixture of argon and oxygen to limit the surface oxidation of platinum. After cleaning, the substrates were functionalized by submerging them in a 1–10 mM solution of 1-octadecanethiol (98%, Sigma-Aldrich) in ethanol. The samples were submerged for up to 24 h to allow the monolayer to reach saturation. The functionalized substrates were cleaned in an ultrasonic bath to remove any octadecanethiol aggregates from the surface before further processing.

The functionalized substrates were patterned by exposing them in a UV-ozone cleaner (PSD PRO-UV T6, Novascan) at 30 °C. During the exposure, deep ultraviolet (DUV) generated reactive oxygen species react with thiol groups in the exposed monolayer to form sulfonate groups.^16,29^ Substrates exposed through a shadow mask were covered with a Si substrate resting directly on the functionalized surface of the substrate, while substrates exposed through a photomask were covered by a DUV transparent quartz photomask with chrome patterning (JD Photodata). The masks were supported by spacers of equal thickness as the exposed substrate, leaving the mask in soft contact with the substrate. The USAF 1951 pattern (see Figure S1 in Supporting Information) was used to test the resolution of the selective deposition over a large range of feature sizes. After exposure, the substrates were submerged in ethanol for 5 min to desorb the oxidized octadecanesulfonate in the exposed regions.

The aqueous barium titanate precursor solution was prepared as described by Raeder et al.^14^ Barium nitrate (0.3 M) and titanium citrate (0.6 M) solutions were prepared separately and combined to a stoichiometric BaTiO_3_ precursor solution with a final concentration of 0.2 M. See Supporting Information for more details. The precursor solution was deposited on patterned substrates by spin coating at 3000 rpm for 50 s. The films were subsequently dried for 4 min at 200 °C. Dried films were heat treated in an AW610 rapid thermal processing (RTP) unit (Allwin) to 450 °C for 2 min in an oxygen atmosphere to remove the organic components and were then heated again to 800 °C for 3 min. All ramp rates were set to 40 °C/s to avoid the formation of intermediate carbonate phases.^30,31^ A heat-treated SAM sample used for XPS analysis was prepared by treating a pristine SAM using the same heating program as was used for annealing the CSD films. The nonpatterned sample used for XRD analysis was prepared by separately spin coating and annealing 4 layers of BTO precursor solution using a custom high temperature heating plate.^32^ Each layer was dried at 200 °C, the same as the other films, but was heated to 365 °C at 100 °C/min, then slowly heated to 435 °C at 50 °C/min to burn out any organics, and finally heated further to 800 °C at 20 °C/s and annealed for 10 min before cooling to room temperature at 100 °C/min. The full process is represented schematically in Figure 1, including the sample selection for XPS.

**Figure 1:**
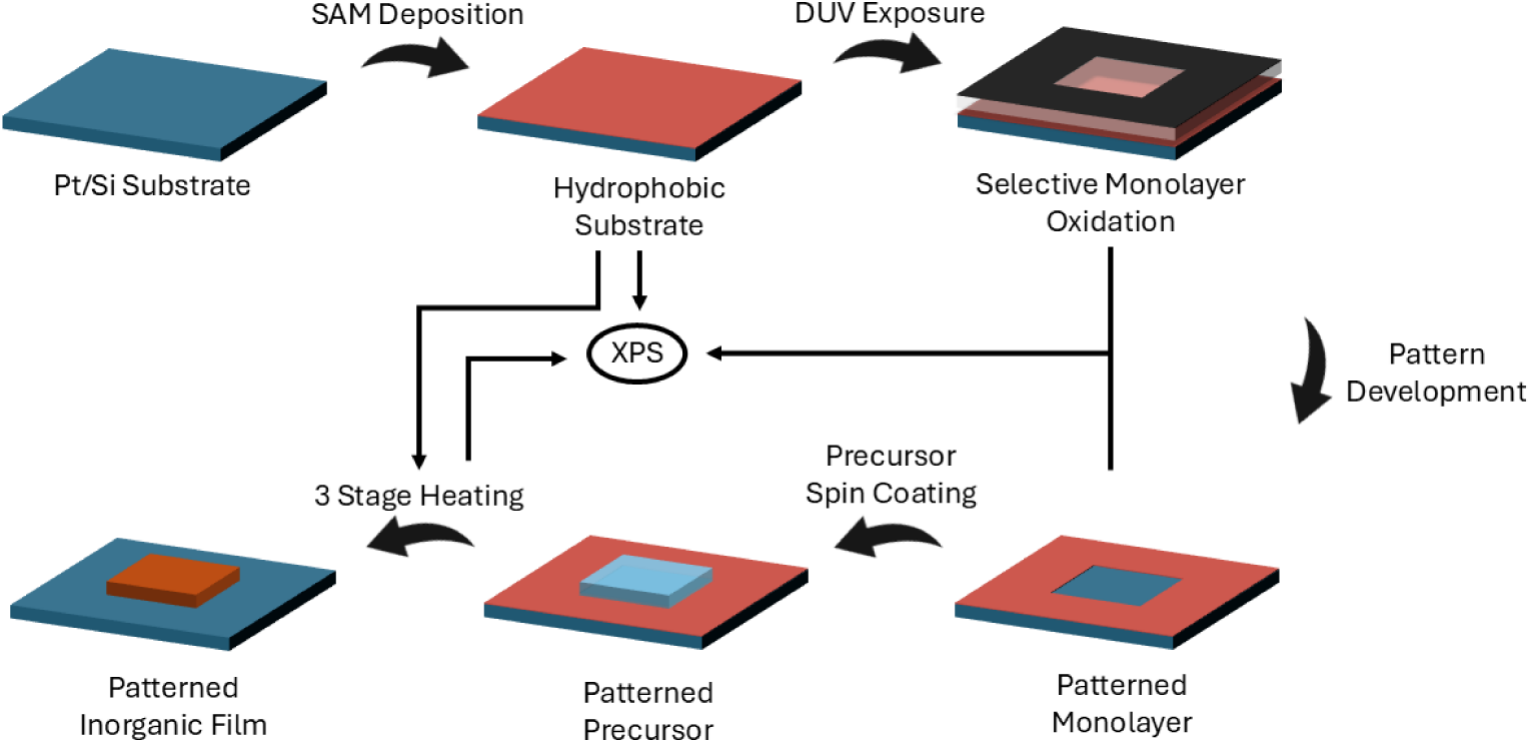
Schematic representation of the selective aqueous chemical solution deposition process. The straight arrows indicate at what points in the process XPS analysis was performed.

### 2.2 Characterization

The hydrophobicity of functionalized and exposed substrates was tested using drop shape analysis (KRÜSS DSA100). As the drop shape analyzer is not set up to use custom liquids, the measurements were performed using water droplets instead. The water contact angle measurements were compared to measurements using manually deposited precursor solution droplets to confirm that was a suitable substitute (see Figures S2–S4, Supporting Information). The water contact angle was measured over 10 s with a 1 s interval to account for spreading effects. The contact angle was determined in three different regions of the substrate to average out any nonuniformities. Patterned samples were mapped using 3D optical profilometry (Bruker Contour GTK) in a combined vertical scanning and phase-shifting interferometry mode (VXI). Individual features were additionally imaged using scanning electron microscopy (SEM, FEI Apreo). Samples were sputter coated with 10 nm of Pt/Pd alloy before SEM imaging. Synchrotron grazing incidence XRD analysis was performed at the BM01 beamline of Swiss-Norwegian beamlines (SNBL) at the European Synchrotron Radiation Facility (ESRF). Samples were analyzed using 0.72932 Å wavelength X-rays at a grazing incidence angle of 2°. A Dectris Pilatus3 × 2 M detector was used to record 2D XRD patterns of the samples, which were then integrated along the azimuth to acquire high quality diffractograms.

X-ray photoelectron spectroscopy (XPS) measurements were conducted using a Kratos Ultra DLD spectrometer equipped with a monochromatic Al Kα source (1486.6 eV), a hemispherical electron energy analyzer, and a magnetic lens to enhance sensitivity. All measurements were performed under ultrahigh vacuum conditions (10^–9^–10^–10^ Torr) with a spot resolution of ≤15 μm. The spectra were calibrated by setting the Au 4f_7/2_ reference peak at 84 eV. Survey and high-resolution spectra were acquired at pass energies of 160 and 20 eV, respectively. For each sample, spectra were collected from three independent spots and subsequently summed to improve the signal-to-noise ratio. Elemental core-level spectra were analyzed using CasaXPS software,^33^ where peaks were fitted with a convolution of Lorentzian and Gaussian line shapes. The background was modeled using a linear combination of Shirley and Tougaard functions, with coefficients optimized simultaneously with the peak fitting. The Pt 4f signal was treated with asymmetric peak profiles to account for multiplet splitting. The minimum number of components required to achieve unstructured fit residuals was used.

## 3 Results

The degree of functionalization of the substrates measured through the water contact angle is summarized in Table 1. The nonfunctionalized, acetone cleaned platinum surface was measured to have a contact angle of 57°. This angle significantly increased after submersion in the 1-octadecanethiol solution, as demonstrated in Figure 2a, reaching a saturation value around 110° after submerging for 24 h in a 5 mM solution. This value matches the expected value of octadecane SAMs.^1^ The surface treatment of the substrates was found to play a large role in the reaction between 1-octadecanethiol and platinum. The water contact angle of a functionalized substrate increased from 59° to 98° when the substrate was cleaned with a 1:1 oxygen/argon plasma instead of pure oxygen plasma before deposition. The lower contact angle for the oxygen plasma cleaned substrate, indicative of a lower SAM density, is likely caused by the formation of platinum oxide during the cleaning process, which inhibits the reaction of thiol groups on the surface.^34^ Cleaning with an oxygen/argon plasma reduces the oxidizing power of the plasma, resulting in improved monolayer deposition.

**Figure 2:**
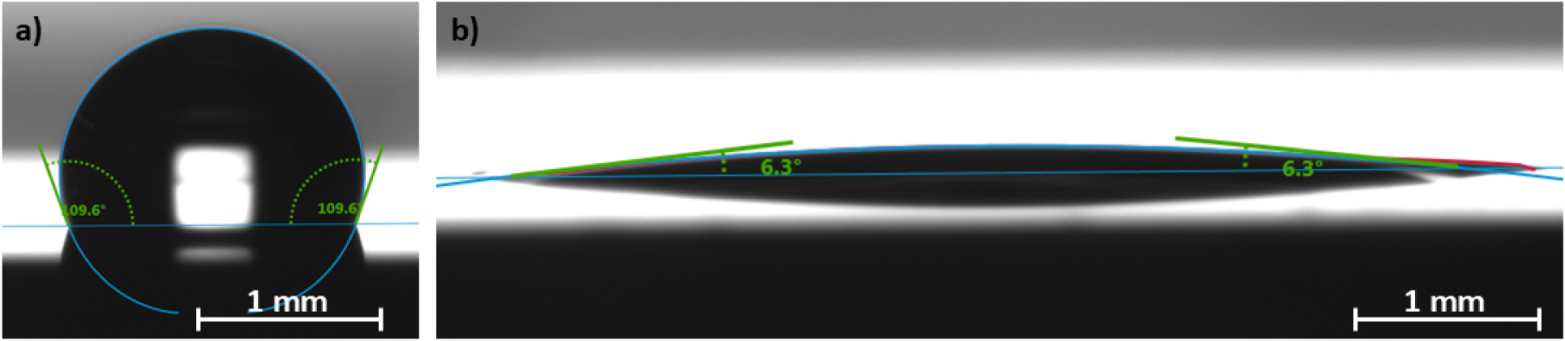
Water contact angle of Pt/Si substrates after (a) 24 h 1-octadecanethiol functionalization and (b) a subsequent 20 min DUV exposure.

**Table 1:** Concentration of 1-Octadecanethiol Solution, Deposition Time, Surface Preparation of the Pt/Si Substrates and Measured Contact Angle after Deposition of 1-Octadecanethiol on Pt/Si Substrates

Concentration (mM)	Deposition time (h)	Surface Preparation	Contact Angle (°)
-	-	Acetone wash	57 ± 3
1	24	Oxygen/argon plasma	100 ± 2
5	24	Oxygen/argon plasma	108 ± 1
10	24	Oxygen/argon plasma	111 ± 1
10	20	Oxygen plasma	59 ± 5
10	20	Oxygen/argon plasma	98 ± 1

Exposure of the functionalized substrates to DUV light lowered the water contact angle to <10° after a 20 min exposure, as shown in Figure 2b. This is well below the contact angle of solvent cleaned platinum, which can be attributed to the oxidation of not only the thiol groups in the monolayer but also the platinum substrate, resulting in a hydrophilic surface. As such, a water contact angle contrast of over 100° was reached between the SAM functionalized surface and the DUV exposed surface. When covered with a shadow mask, the contact angle of the monolayer remained unchanged after UV exposure, confirming that the monolayer is only removed in regions that are exposed directly to the UV-generated reactive oxygen species.

The wetting selectivity of the BTO precursor solution during spin coating was tested on functionalized substrates exposed through a simple shadow mask covering half of the substrate. Profilometry of the film deposited after 20 min of DUV exposure shows high selectivity of the precursor solution for the exposed region of the substrate. However, some dewetting defects are present in the boundary region, as highlighted in Figure 3a. After increasing the DUV exposure time to 25 min, these defects disappear, resulting in a fully covering film with a sharp boundary, as shown in Figure 2b. The thickness of the deposited films is approximately 0.1 μm, regardless of the exposure time.

**Figure 3:**
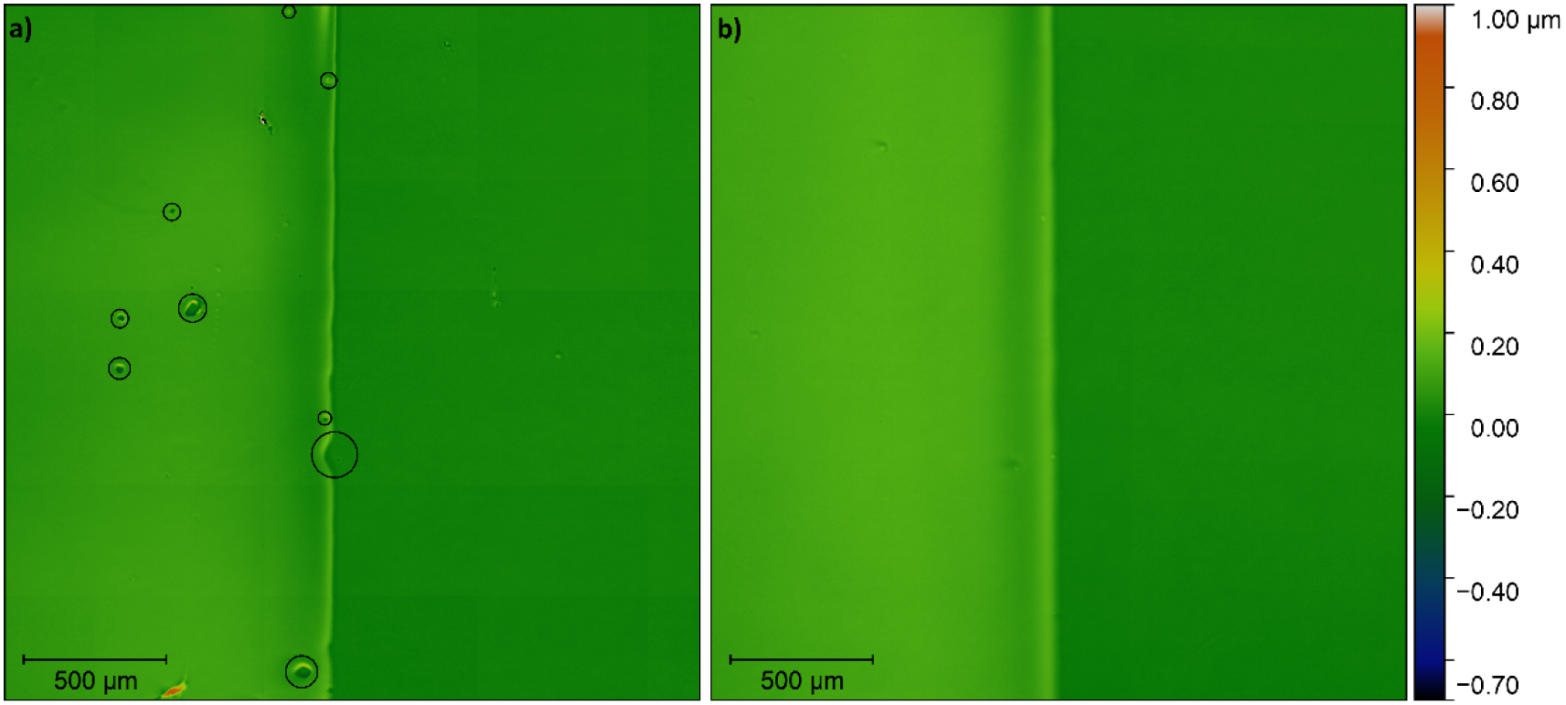
Height profiles of dried BTO precursor films deposited on substrates exposed for (a) 20 and (b) 25 min through a shadow mask covering the right half of the substrate. Dewetting defects are circled in black.

Pt 4f and S 2p XPS spectra of samples after SAM deposition, maskless DUV exposure, washing of the exposed SAMs, and after a full RTP heating cycle on a pristine SAM are shown in Figure 4. The assignments of the fitted peaks are summarized in Table 2. The Pt 4f spectrum of the pristine SAM shows multiple oxidation states of Pt, indicating a partially oxidized surface comprised of metallic Pt, PtO, and PtO_2_. This lines up with our expectations after cleaning the surface with a mixed O_2_/Ar plasma treatment. The S 2p spectrum contains two peaks at 162.93 and 163.93 eV, associated with surface bound and unbound thiol groups, respectively. The large amount of unbound thiol molecules present may indicate multilayer SAM deposition; however, this may also be attributed to unbound molecules trapped in the monolayer that cannot be removed using ultrasonic treatment or UHV evacuation. After DUV exposure, the Pt 4f spectrum maintains the mixed composition of various oxidation states, indicating partial oxidation of Pt. This may indicate that the surface oxidation of Pt is not strongly affected by DUV exposure; however, the surface oxidation may also have partially degraded during evacuation in UHV, resulting in a lower surface oxide signal during the measurement.^35^ The S 2p signal shows a full conversion of sulfur from thiol to the higher oxidation state associated with sulfonate groups, confirming complete oxidation of the monolayer. The S 2p spectrum for the sample measured after washing in ethanol does not differ significantly from the sample measured after DUV exposure, indicating that some sulfonate groups remain on the surface after dissolution. The S/Pt atomic ratios were calculated from the relative peak areas, allowing for the comparison of the relative amount of sulfonate groups on the samples before and after dissolving the sulfonates in EtOH. Before dissolution, the S/Pt ratio was 0.106, while after dissolution it was 0.081, indicating a 23% decrease in sulfur content. After annealing, the Pt 4f spectrum shows a single component matching the Pt^2+^ state of PtO, indicating significant surface oxidation covering the full penetration depth of the X-rays due to annealing in oxygen atmosphere. The S 2p spectrum shows no sulfur signal, indicating that the SAM was fully decomposed during the annealing procedure.

**Table 2:** Peak Assignments for the Pt 4f and S 2p XPS Spectra of 1-Octadecanethiol SAMs in Pristine Condition, after a 25 min DUV Exposure, after Exposure and a 5 min Soak in EtOH, and after Heating for 3 min at 800 °C in O_2_ ^a^

		Pristine SAM	DUV exposed	Soaked in EtOH	Heat-treated
Pt 4f_7/2_	Metallic Pt (◆)^36^	70.98	70.98	70.98	-
	Pt^2+^ (PtO) (▲)^36^	72.22	72.05	72.06	72.26
	Pt^4+^ (PtO_2_) (●)^36^	74.35	74.26	74.25	-
S 2p	Metal bound thiol (◆)^34^	162.93	-	-	-
	Unbound thiol (△)^34^	163.93	-	-	-
	Sulfonate (○)^34^	-	167.76	167.66	-

a All peak positions are listed in eV.

**Figure 4:**
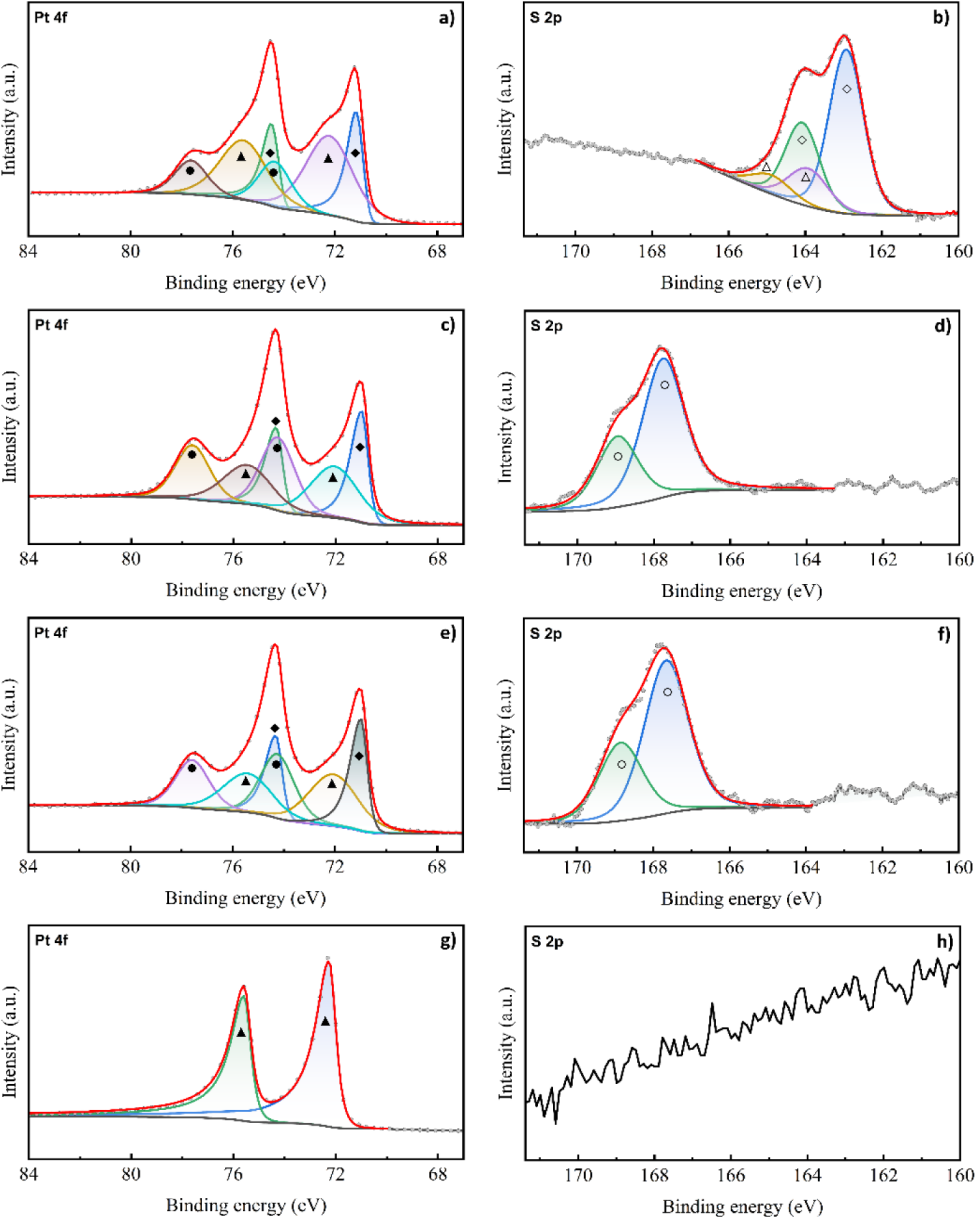
Pt 4f and S 2p spectra of samples with a pristine SAM (a, b), a SAM exposed to DUV light for 25 min (c, d), a SAM soaked in EtOH for 5 min after DUV exposure (e, f), and a SAM annealed at 800 °C in oxygen for 3 min (g, h). The red line shows the fitted data used for peak analysis. Assignment of the fitted peaks can be found in Table 2.

After optimizing the exposure procedure for maximum selectivity during spin coating, functionalized substrates were exposed to DUV light through a quartz photomask to allow for the deposition of more complex structures and investigate the resolution that can be achieved. After a 25 min DUV exposure through the photomask, films deposited on the patterned substrate show an excellent transfer of the mask pattern. The precursor has high selectivity for the exposed regions, even when depositing smaller structures. Figure 5a shows the smallest feature to maintain its shape in the pattern. The measured average width of the three lines is 29.0 ± 2.0 μm, which is in good agreement with the pattern width of 27.8 μm. The smallest recognizable feature is a series of three vertically aligned dots, shown in Figure 5b. Features with dimensions below 15 μm did not display successful deposition. Table 3 compares the feature dimensions of the quartz photomask with BTO precursor samples prepared in triplicate. The feature dimensions on the samples match both each other and the photomask, indicating both replicability of the achieved dimensions and fidelity between the photomask and the deposited features. While the 15.6 μm feature was successfully deposited on sample 3, its dimensions could not be measured as capillary bridges made it impossible to identify the individual structures.

**Table 3:** Comparison of Feature Dimensions of Selectively Deposited BTO Precursor Solutions and the Photomask^a^

Mask	Sample 1	Sample 2	Sample 3
140.3	140 ± 3	139 ± 3	129 ± 4
99.2	97 ± 3	97 ± 4	96 ± 3
24.8	26 ± 6	23 ± 3	28 ± 4
15.6	16 ± 3	15 ± 2	-

a All dimensions are listed in μm.

**Figure 5:**
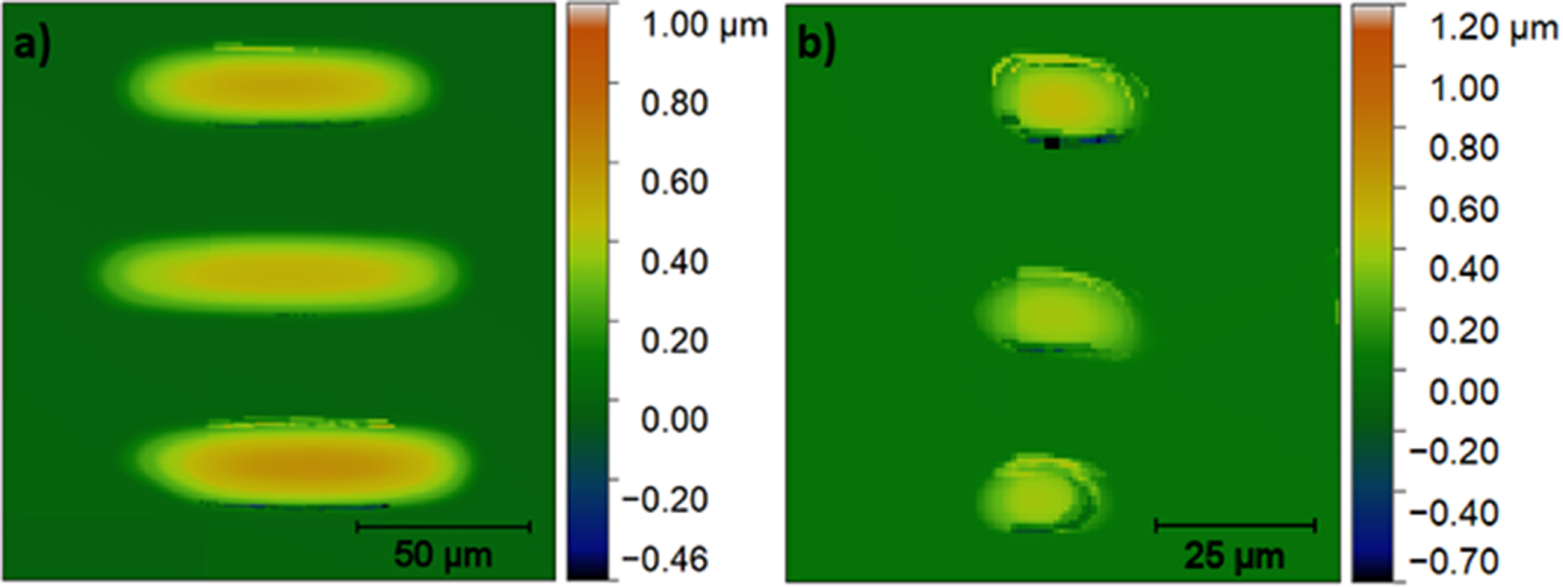
The Smallest dried BTO precursor features selectively deposited after a 25 min DUV exposure, with a line width of (a) 29.0 ± 2.0 μm and (b) 15.0 ± 2.0 μm.

The thickness of the deposited features ranges from 100 nm to over 2 μm, with height profiles varying significantly between features. Features below 70 μm in width have a near symmetrical profile, but larger features have a significant thickness gradient, increasing in thickness along the direction of flow during the spin coating process. Figure 6 demonstrates the difference in height profiles between a 139 ± 3 μm and 56 ± 4 μm feature. This indicates that the flow behavior is not only affected by the confinement of the solution, but also the dimensions of the deposited features. Full profilometry scans of the patterned films are presented in Figures S5 and S6 in the Supporting Information. SEM images of the deposited structures (Figure 7a,c) agree with corresponding profilometry images (Figure 7b,d), confirming the geometry of the deposited structures. Figure 7a,c shows the smooth, curved surface of the deposited structures, and Figure 7c also clearly demonstrates the formation of a capillary bridge between two features where the centrifugal force during spin coating was insufficient to overcome the surface tension of the solution.

**Figure 6:**
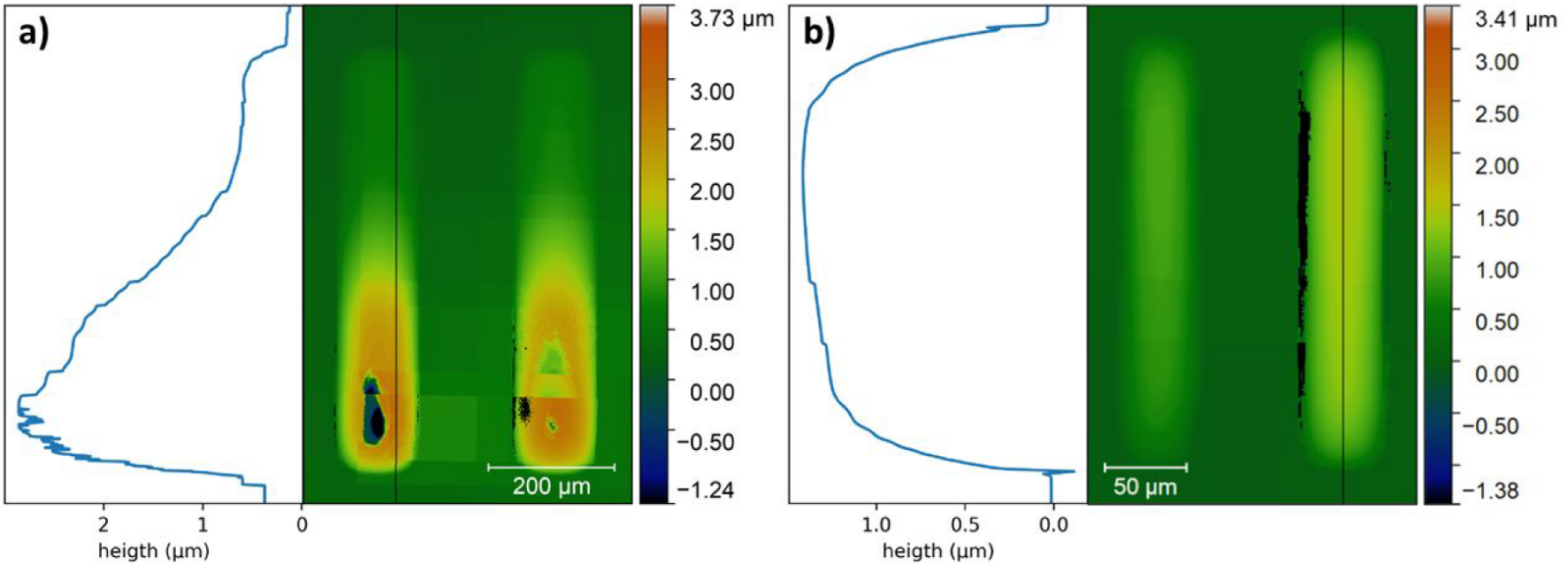
Profilometry scans with matching height profiles of (a) 139 μm and (b) 56 μm dried BTO precursor features as deposited. The features were deposited after a 25 min DUV exposure. The height profiles were recorded at the indicated lines.

**Figure 7:**
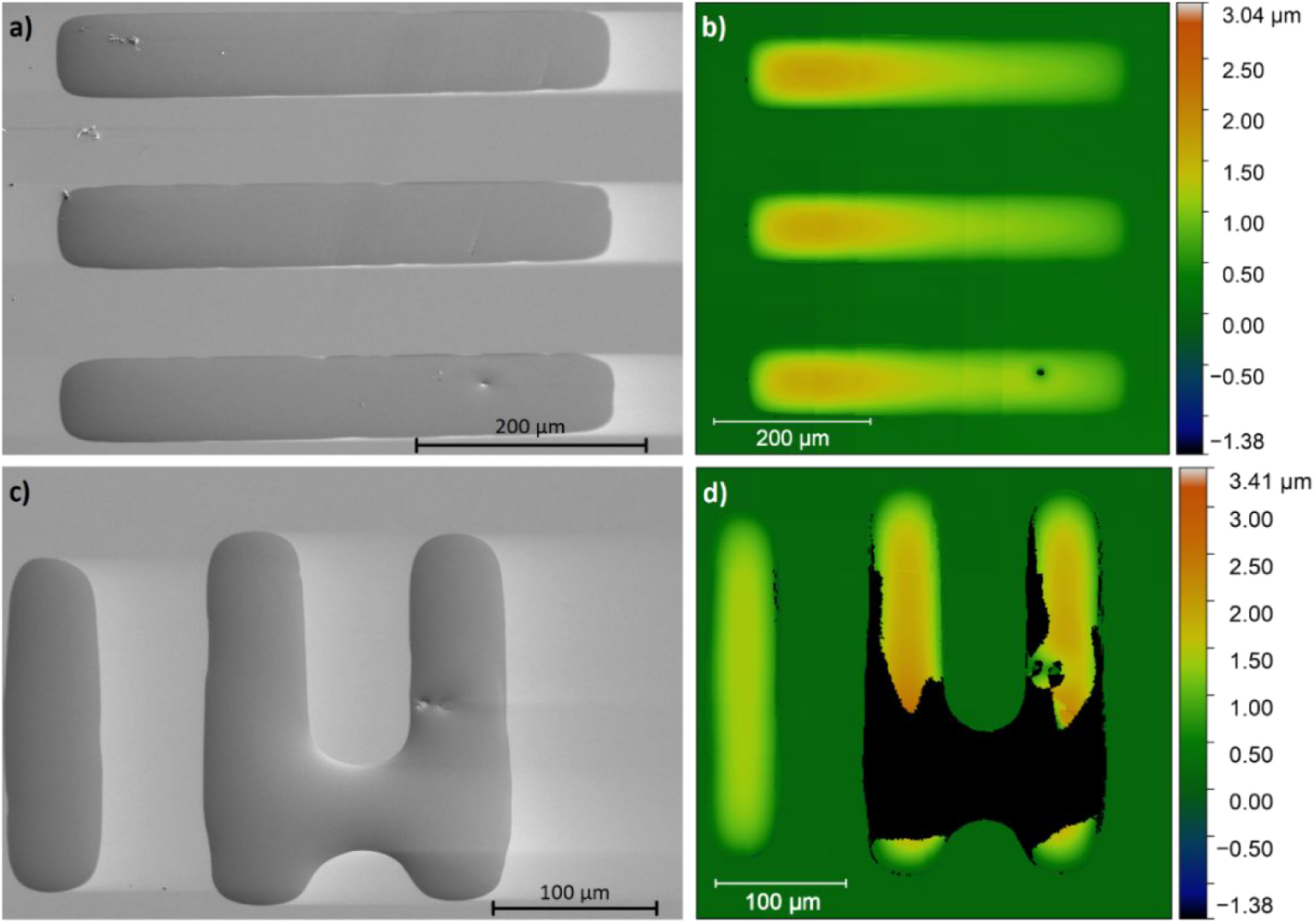
SEM images (a, c) and profilometry profiles (b, d) of as-deposited features with a width of 99 μm (a, b) and 63 μm (c, d). The features were deposited after a 25 min DUV exposure. Lighter regions near the deposited features on the SEM images are charging artifacts.

A comparison of the dried as-deposited and annealed films (Figure 8) shows a significant decrease in film thickness after annealing. The in-plane dimensions of the annealed structures match well with the as-deposited structures, and the height variations present before annealing remained present in the annealed structures. This shows that aside from the expected decrease in height due to the removal of the organic components, the dimensions of the structures are unaffected by the annealing process.

**Figure 8:**
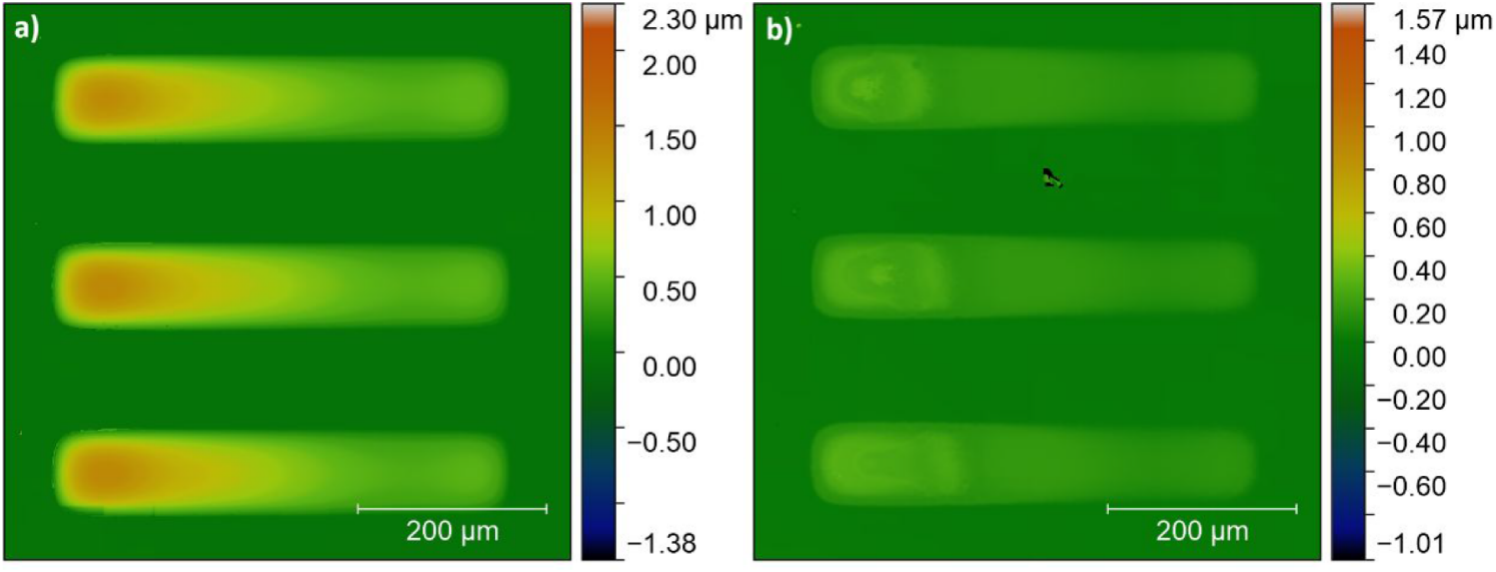
Optical profilometry images of a selectively deposited film of (a) as-deposited BTO precursor and (b) annealed BTO films. The annealed film was held at 450 °C for 2 min, then at 800 °C for 3 min.

SEM imaging of the annealed films clearly shows the dense granular structure of the deposited films, as is typical of crystalline thin films. There is no clear grain structure visible on the surface or in the cross-section, indicating that the grains are small. A close up of a 150 nm thick region of the film, as seen in Figure 9a, shows gaps between the grains of approximately 100 nm. The formation of such gaps has previously been observed in films deposited by aqueous CSD.^14,30^ Cross-section SEM (Figure 9a, inset) shows a dense film with no preferential growth direction on the substrate. The cross-section SEM also did not show any evidence of pinholes in the film, indicating that the voids observed in the surface morphology may be confined to the surface. SEM imaging of a 190 nm thick region, as seen in Figure 9b, showed the initiation of cracks that formed during annealing.

**Figure 9:**
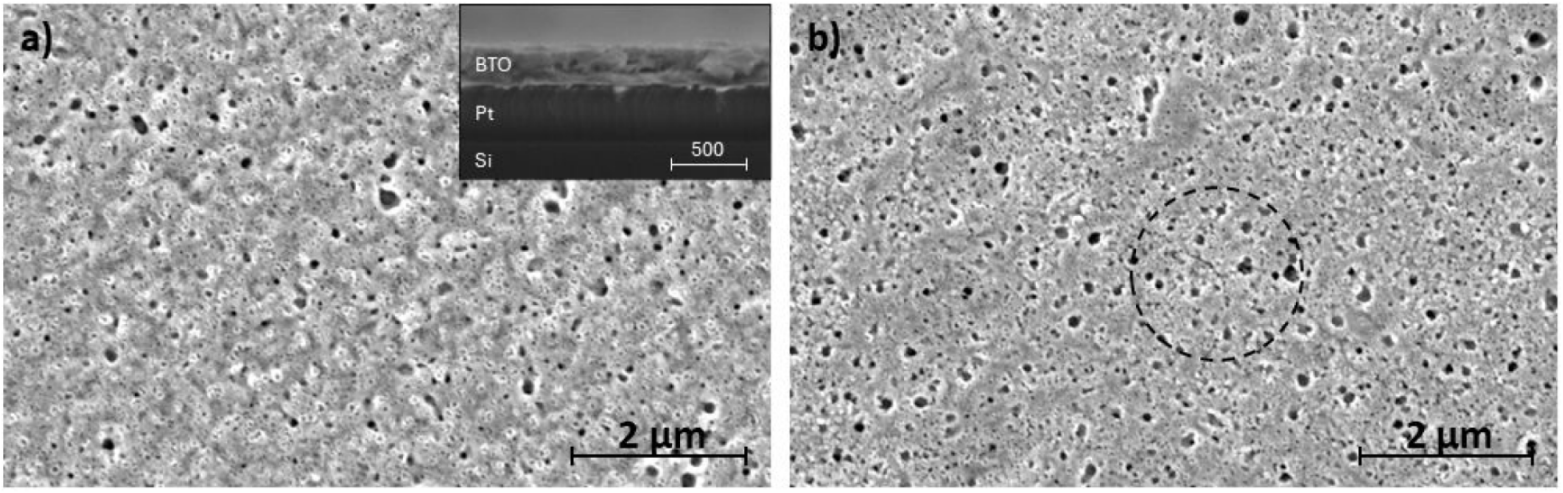
SEM images of a selectively deposited BTO film annealed at 800 °C with a thickness of (a) 150 nm with a cross-section image of a 240 nm region (inset), and (b) 190 nm, with a crack initiation site circled in red. The scale bar in the inset is in nm.

Grazing incidence XRD analysis of an annealed BTO film in Figure 10, shows peaks associated with a number of high symmetry axes of tetragonal BTO, indicating polycrystallinity, as expected on a Pt substrate. A minor peak associated with the BaCO_3_ intermediate phase was identified at 13.4°,^37^ indicating that the film is not phase pure.

**Figure 10:**
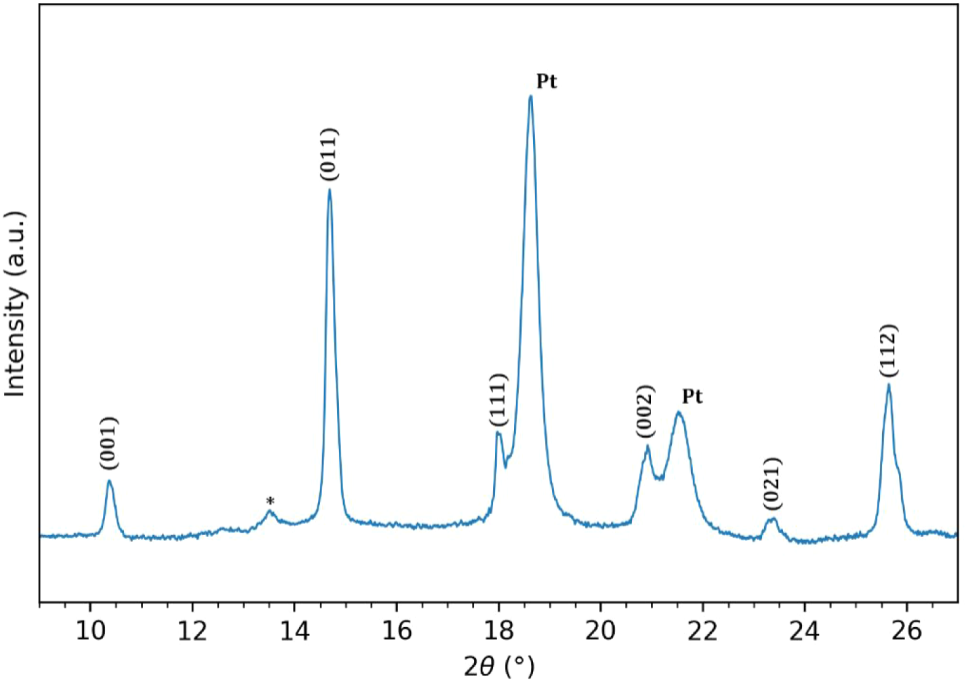
GI-XRD pattern of a BTO film annealed at 800 °C measured using 0.72932 Å synchrotron radiation. All BTO diffraction lines (PDF 00-071-0482) are labeled with their respective index. Platinum substrate peaks (PDF 00-004-0802) are labeled “Pt”. The intermediate BaCO_3_ phase is labeled ‘*’.^37^

## 4 Discussion

Figures 3 and 6 show that a patterned monolayer of functionalized SAMs can be used as a mask for the selective deposition of thin films from aqueous precursor solutions. With careful tuning of the surface properties, a minimal feature size of 15.6 μm has been achieved, which is promising for microscale applications and is competitive with other vacuum-free bottom-up patterning techniques such as inkjet printing,^38^ which has a minimum line size of 20–30 μm. Table 4 shows a comparison of the resolution achieved by CSD on a UV patterned SAM and comparable techniques. The minimal feature size, high scalability, and simplicity of the process, make this a promising technique for industrial applications, while the improved material efficiency and minimal use of vacuum and high energy equipment allow for sustainable processing. Additionally, Figure 4 shows that the deposited features have a unique profile that appears to be the result of spin coating on an inhomogeneous surface.

**Table 4:** Minimum Feature Size Achieved by Various Oxide Thin Film Patterning Techniques

Technique	Minimum feature size (μm)
Mask aligned litho-etch	1 ^39,40^
Microcontact printing	2 ^41^
Inkjet printing	20 ^38^
SAM-based selective deposition	15.6

The resolution limit that was reached appears to be caused by the complete dewetting of features below the resolution limit. Most likely, as the spacing of hydrophobic and hydrophilic regions becomes very small, the solution stops interacting with them as discrete regions, and instead treats them a single, chemically inhomogeneous surface. As described by Cassie’s law,^42^ a droplet on such a surface will behave as if the surface has an average wettability weighted by the relative surface areas of the different regions. If this average wettability is sufficiently poor, the solution dewets the entire region, which would explain why no features were deposited below 15 μm. A loss of resolution in the patterning of the SAMs, either due to diffusion of the hydrophobic monolayer or underexposure of the features, might exacerbate this effect, but cannot be solely responsible, as no significant reduction of dimensions was observed in larger features.

From the results of the SAM functionalization, the degree of oxidation of the platinum substrate had a large influence on the deposition of the 1-octadecanethiol SAM. During deposition, it was found that a surface oxide layer inhibited the formation of SAMs on platinum, which matches previous reports that oxidizing surface preparation methods lead to poor thiol deposition.^34,43^ This has been hypothesized to be the result of the greater bond strength of the Pt–O bond compared to the Pt–S bond.^34^ Reducing the oxidation power during the substrate preparation was critical to reduce the formation of platinum oxide on the substrate surface and successfully deposit SAMs on the substrates, even when using long deposition times.

XPS analysis confirmed that a thiol-based coating is chemically bound to the Pt substrate, confirming the successful deposition of the 1-octadecanethiol SAM. In addition to thiol groups bound to Pt, the S 2p spectrum of a pristine SAM (Figure 8b) also showed a significant amount of thiol groups not bound to Pt. This could indicate that physisorbed thiol molecules remain on the sample in a multilayer stack on top of the monolayer. However, Petrovykh et al.^34^ observed similar results even after aggressive cleaning procedures intended to remove any unbound thiol molecules, indicating that this signal does not necessarily originate from thiol molecules physisorbed on the sample surface. They hypothesize that the chemical shift relative to the bound thiol molecules may be caused by chemical binding to oxidized instead of metallic Pt. As the Pt 4f spectrum (Figure 8a) shows partial oxidation of the platinum surface, this would also explain the two different binding energies of S found in our sample. After a 25 min DUV exposure, the XPS analysis shows a full conversion of S from thiol to sulfonate groups, confirming complete oxidation of the monolayer. Washing of the monolayer in EtOH was not sufficient to remove all oxidized sulfonate groups from the substrate, and in fact only a 23% decrease in S was detected between the exposed and dissolved monolayers. However, as the exposed sulfonate groups are not strongly bound to Pt, sulfur may have evaporated from the exposed sample during evacuation in UHV, reducing the amount of S measured on the DUV exposed sample. As a result, the difference in S content measured using XPS before and after washing in EtOH may not accurately reflect the actual decrease in exposed SAM molecules on the sample as a result of the washing procedure. As such, the quantification of the XPS data is not necessarily representative of the samples as processed. After the heat treatment, there was no S 2p signal, confirming the decomposition and volatilization of the SAM, leaving no detectable sulfur contamination on the surface. The 2-stage heating profile used during the annealing of the BTO films has been designed such that the decomposition and volatilization of the organic components occur before the nucleation of inorganic components. As such, the presence of oxidized SAM molecules on the patterned substrate is not expected to impact the annealing process of the deposited film or any future processing steps, though an in-depth in situ XRD study of the BaTiO_3_ nucleation process is required to confirm this.

The presence of asymmetrical height profiles of the deposited structures, and the dependence of this asymmetry on the size of the deposited structure shows that the dynamics of the spin coating process on a patterned substrate differ significantly from a homogeneous substrate, as the same asymmetry is not observed in the films covering half the substrate, as shown in Figure 2. Additionally, the maximum thickness achieved for the patterned structures is over 20× that of the covering films. It should be noted that, since the SAM is less than 3 nm thick, the monolayer’s contribution as a physical flow barrier is negligible. Rather, the flow resistance imparted by the monolayer can be considered entirely chemical due to its low wettability. Regardless, this flow barrier appears to cause a buildup of solution at the border between hydrophilic and hydrophobic regions along the spin coating direction. The slope this creates in the height profile of the deposited structure increases with increasing dimensions of the deposited structure, indicating that this behavior does not only depend on the presence of a flow barrier, but also on the dimensions of the structure in which the solution is confined. As such, the flow behavior of a solution spin coated on a substrate that is chemically inhomogeneous on a microscopic scale appears to be much more complex than previously anticipated.

The change in the spin coating dynamics affects not only the height profile of the deposited features, but also the morphology of the annealed films. From the SEM images of the annealed films, cracking occurred in regions of the film with a thickness of 190 nm and above. This indicates that the critical thickness for crack formation in the film is 190 nm.^44^ The cracking is most likely caused by the drying of the film to form an amorphous film. As the film dries, capillary pressure builds up pressure gradients across the film, resulting in the formation of cracks.^45^ The critical thickness for this type of cracking in oxide-based films usually lies around 100–300 nm,^46–48^ which matches the observed critical thickness. Stress introduced during crystallization was also considered as a source of cracking. However, this mechanism was considered to be an unlikely source, as the grain size is much smaller than the film thickness, allowing for strain relaxation along the grain boundaries.

XRD analysis of an annealed film shows that the films are polycrystalline, as expected on a Pt/Si substrate. The weak BaCO_3_ diffraction peak observed at 13.4° belongs to a previously detected intermediate phase in BTO produced through solution-based synthesis.^31,36^ Bakken et al.^49^ indicate that a Pt/Si substrate may stabilize the intermediate phase compared to oxide substates. Bakken et al. also reported that the formation of the intermediate phase can be prevented from forming by tuning the annealing process, such as by increasing the ramp rate to the annealing temperature, or by increasing the annealing temperature. Preventing the formation of the carbonate intermediate phase is of additional importance during selective deposition of BTO, as the increased film thickness increases the diffusion path length of CO_2_ from the decomposition of the carbonate. As such, selective deposition may further encourage the formation of the carbonate phase.

Further study of the spin coating dynamics on a chemically inhomogeneous surface is required to improve the uniformity of the deposited structures. The height uniformity and overall thickness of the deposited structures deviated significantly from films deposited under the same conditions on homogeneous substrates. As such, spin coating protocols developed for nonpatterned films cannot be applied directly to spin coating on patterned substrates. By optimizing the spin coating process for patterned deposition, we expect to achieve height profiles of deposited structures closer to those of covering films. This will not only improve the thickness uniformity of the annealed structures, but also the morphology of the structures, as the formation of cracks is suppressed.

## 5 Conclusions

Patterned hydrophobic SAMs have successfully been used to selectively deposit a micropatterned BTO film using aqueous CSD. The patterned substrate has a water contact angle contrast of over 100°, which results in a high selectivity of the solution for the hydrophilic regions. The achieved feature size of 15.6 μm has potential for applications such as capacitors and is competitive with other bottom-up patterning techniques of solution-based films. Careful modification of the surface properties not only on the SAM functionalized regions but also the platinized substrate itself was determined to be key in the successful deposition of both the SAM and the patterned precursor film. The fluid dynamics of spin coating on a patterned substrate, as observed in the dewetting of small features and the size dependence of the height profile, are not yet fully understood but have been demonstrated to be significantly more complex than on a homogeneous substrate. In its current state, this novel fabrication process has been demonstrated to be an effective way of selectively depositing oxide films using aqueous CSD on Pt/Si substrates, although it may be adapted for other materials that can be deposited from aqueous solutions. Furthermore, by adapting this technique for silane-based SAMs, this process may be expanded in the future to cover more substrate compositions, offering a sustainable and highly scalable alternative to litho-etch thin film patterning for a wide range of applications.

## Supplementary Material



## Notes

USAF 1951 test pattern; BaTiO_3_ precursor preparation; optimization of DUV exposure; full profilometry scans (PDF)
