# Supplementary material for: Selective Aqueous Chemical Solution Deposition Using Patterned Self-Assembling Monolayers
Source: ACS Omega. 2026 Mar 30;11(13):20369–79. doi: 10.1021/acsomega.5c11372 (PMC13063017; doi:10.1021/acsomega.5c11372)
Supplement: Supplementary file 1 [file ao5c11372_si_001.pdf]

Selective Aqueous Chemical Solution Deposition using Patterned Self-Assembling Monolayers

Karola Neeleman*, Hung Quoc Nguyen, Daniel Rettenwander, Julia Glaum and Mari-Ann Einarsrud*

Department of Materials Science, NTNU Norwegian University of Science and Technology, 7034 Trondheim, Norway

***Corresponding authors**

Supporting Information

USAF 1951 resolution test pattern

The USAF 1951 resolution test pattern consists of sets of three horizontal and vertical bars with equal spacing to their width. The pattern progressively gets smaller as the group and element numbers increase. It is a standard pattern for determining optical resolution by finding the smallest element in which three distinct horizontal and vertical bars can be identified. Pattern adapted from the USAF 1951 GDSII design by Hsieh-Fu Tsai [1].

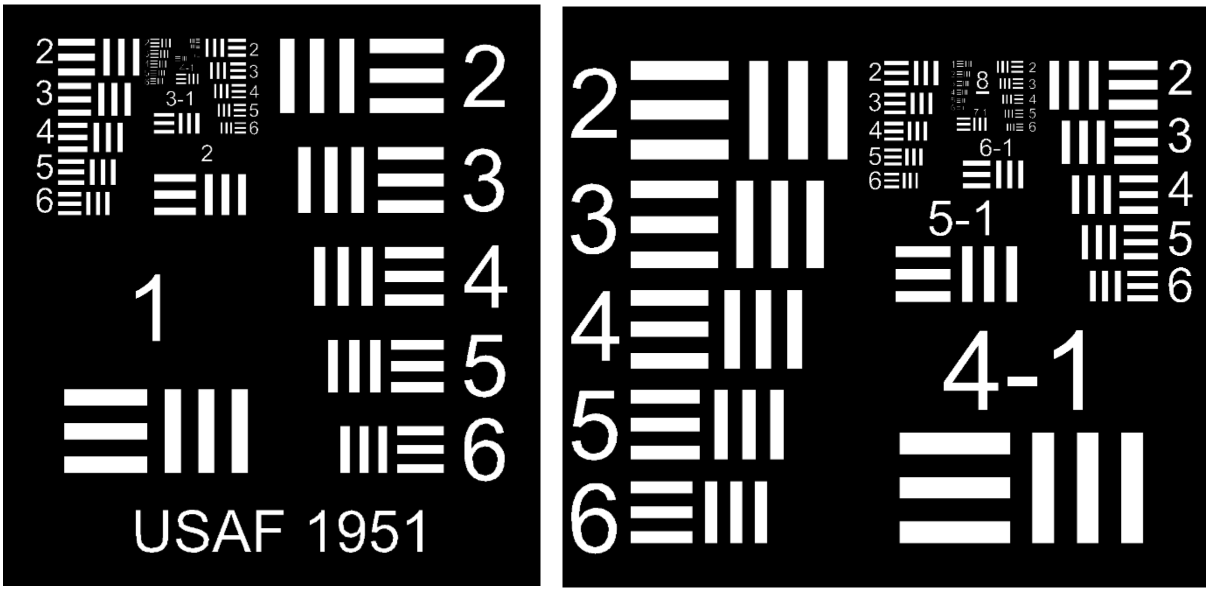

Figure S1: The USAF 1951 mask used in the experiments. Black areas in the design are chrome plated on the mask.

Table S1: Line width of features in the USAF 1951 resolution test pattern in µm.

	Group number							
Element	1	2	3	4	5	6	7	8
1	250	125	62.5	32.25	15.63	7.81	3.91	1.95
2	222.72	111.36	55.68	24.84	13.92	6.96	3.48	1.74
3	198.43	99.21	49.61	24.80	12.40	6.20	3.10	1.55
4	176.78	88.39	44.19	22.10	11.05	5.52	2.76	1.38
5	157.49	78.75	39.37	19.69	9.84	4.92	2.46	1.23
6	140.15	70.15	35.08	17.54	8.77	3.38	2.19	1.10

$BaTiO_3$ precursor preparation

The barium nitrate solution was prepared using $Ba(NO_3)_2$ (Sigma-Aldrich, 99.999 %) dried at 200 °C which was dissolved in an aqueous solution of ethylenediaminetetraacetic acid (EDTA, >98.5 %, Sigma-Aldrich) and citric acid (CA, Sigma-Aldrich, anhydrous, ≥99.5 %) at 60 °C to a molar ratio of 1:1:2 Ba:EDTA:CA. The pH of the solution was adjusted to neutral by the addition of ammonia solution (25-30 %) and finally the solution was diluted to the target concentration of 0.3 M. The titanium citrate solution was prepared by dissolving Ti-4-isopropoxide (Sigma-Aldrich, >97 %) in an aqueous citric acid solution to a molar ratio of 1:2 Ti:CA. The solution was stirred for 12 h at 80 °C before the pH was adjusted to neutral using ammonia solution. The solution was diluted to a target concentration of 0.6 M. The exact concentration of the Ti solution was determined using thermogravimetric analysis. The Ba and Ti solutions were combined in stoichiometric ratio to create a $BaTiO_3$ precursor solutions with a final concentration of 0.2 M.

Optimisation of DUV exposure

While a 20 min DUV exposure of a functionalised substrate resulted in only minor dewetting defects when exposing through a shadow mask, there was a significant dewetting of small features during exposure through a photomask. This was improved by increasing the DUV dosage by 25%, which may indicate that smaller features may require higher wettability to achieve deposition. Alternatively, while the fused silica glass used for the fabrication of the photomask is highly transparent to wavelengths down to 175 nm, some DUV light may still be absorbed. This would result in a longer exposure time being required to reach the same UV dose on the substrate.

Full profilometry scans

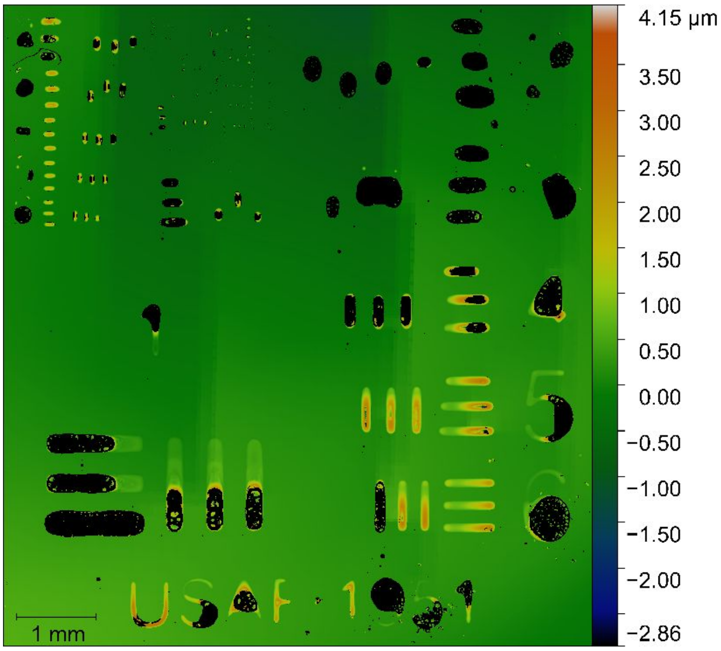

Figure S2: Profilometry image of a BTO precursor film deposited on a functionalised substrate exposed through a quartz photomask for 20 min.

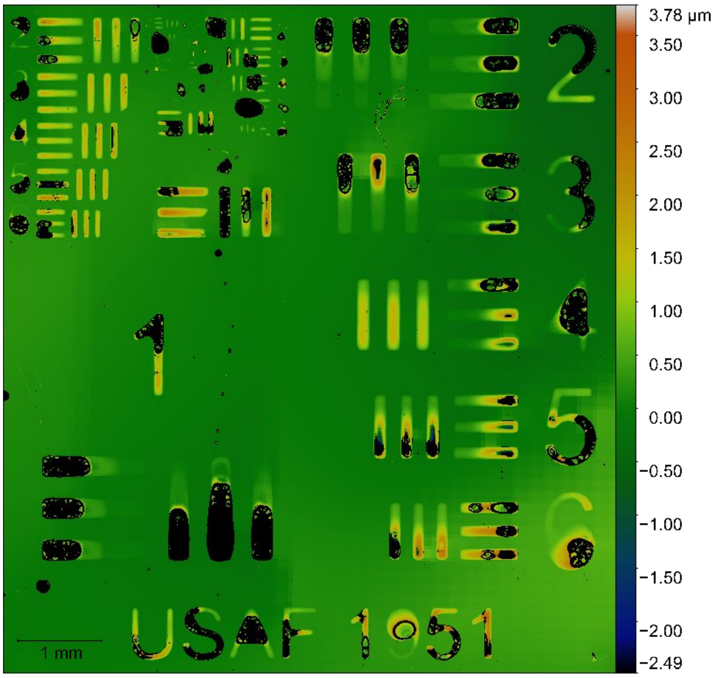

Figure S3: Profilometry image of a BTO precursor film deposited on a functionalised substrate exposed through a quartz photomask for 25 min.

[1] H.F Tsai, "GitHub - oist/resolutiontestingreticle: A resolution testing reticle for 5" chrome mask designed by Hsieh-Fu Tsai." https://github.com/oist/resolutiontestingreticle
